# Conflicting selection pressures on synonymous codon use in yeast suggest selection on mRNA secondary structures

**DOI:** 10.1186/1471-2148-8-224

**Published:** 2008-07-31

**Authors:** Nina Stoletzki

**Affiliations:** 1 Ludwig-Maximilan Universität, Biocenter, Grosshadernerstr. 2, D-82151 Planegg-Martinsried, Germany; 2 Centre for the Study of Evolution, School of Life Sciences, University of Sussex, Brighton BN1 9QG, UK

## Abstract

**Background:**

Eukaryotic mRNAs often contain secondary structures in their untranslated regions that are involved in expression regulation. Whether secondary structures in the protein coding regions are of functional importance remains unclear: laboratory studies suggest stable secondary structures within the protein coding sequence interfere with translation, while several bioinformatic studies indicate stable mRNA structures are more frequent than expected.

**Results:**

In contrast to several studies testing for unexpected structural stabilities, I directly compare the selective constraint of sites that differ in their structural importance. I.e. for each nucleotide, I identify whether it is paired with another nucleotide, or unpaired, in the predicted secondary structure. I assume paired sites are more important for the predicted secondary structure than unpaired sites. I look at protein coding yeast sequences and use optimal codons and synonymous substitutions to test for structural constraints. As expected under selection for secondary structures, paired sites experience higher constraint than unpaired sites, i.e. significantly lower numbers of conserved optimal codons and consistently lower numbers of synonymous substitutions. This is true for structures predicted by different algorithms.

**Conclusion:**

The results of this study are consistent with purifying selection on mRNA secondary structures in yeast protein coding sequences and suggest their biological importance. One should be aware, however, that accuracy of structure prediction is unknown for mRNAs and interrelated selective forces may contribute as well. Note that if selection pressures alternative to translational selection affect synonymous (and optimal) codon use, this may lead to under- or over-estimates of selective strength on optimal codon use depending on strength and direction of translational selection.

## Background

Messenger RNA (mRNA) sequences encode the amino acid sequence of the protein but may also bear additional information. For example, certain synonymous codons may improve translation [1-3] and a variety of motifs may regulate expression at the level of translation, cellular localization, decay or splicing [4-9]. Many of these motifs are secondary structures, and eukaryotic mRNAs contain regulatory structures in their 5' and 3' UTRs [10-15], or introns [16,17]. However, it remains unclear whether secondary structures in the coding regions are of functional importance. Laboratory studies suggest that local secondary structures within coding regions can interfere with translation [18,19], and one may therefore expect selection against structures that are too stable. Surprisingly, however, several bioinformatic studies find that RNA structures within the protein coding regions are more stable than expected by chance [20-23] (but see [24] for opposing result). These studies used various algorithms to predict the secondary structures of mRNA sequences, and then compared the free energy values of these structures to the values for randomized sequences.

Here, I test for selection on mRNA secondary structure using another approach. Instead of testing for unexpected structural stabilities, I directly compare the selective constraint of sites that differ in their importance for the predicted secondary structure. I.e. I predict the secondary structure of coding yeast sequences using different algorithms, and for each nucleotide, I identify whether it is paired with another nucleotide, or unpaired. I assume paired sites are more important for the predicted secondary structure than unpaired sites. If there is selection for secondary structures, one might expect higher structural constraint at paired than at unpaired sites. Such constraint would affect synonymous codon use and substitution rates. In *S. cerevisiae *a relationship between codon use, tRNA abundance and expression level indicates that codon use is affected by selection for translationally optimal codons [1]. If there is selection for mRNA structure, structurally important sites may be under conflicting selection pressures: a codon might support the preferred mRNA structure that is translationally non-optimal. Under structural selection, one might expect lower numbers of optimal codons at paired than at unpaired sites. If mRNA structure is conserved across species, one might further expect lower numbers of synonymous substitutions at paired than at unpaired sites; possible compensatory substitutions however may make the latter test predictions less clear-cut. When a mutation occurs at a paired site and disrupts the pairing ability, a second compensatory mutation on the corresponding paired site may restore the pairing ability [25,26]. Compensatory mutations may increase substitution numbers at paired sites. Innan and Stephan [27] show however, that unless selection against deleterious intermediates is very small, substitutions should occur only very slowly in paired regions [27].

Accurate structure prediction is obviously crucial for these tests. In several studies [20-22], mRNA structures are predicted by thermodynamic properties using the minimum free energy (MFE) algorithm [28] only although taking the whole ensemble of possible structures and comparative information into account is known to increase predictive accuracy [29-32]. I therefore predict the secondary structures by thermodynamic and comparative information (RNA- and ALIfold [33]), using the minimum free energy (MFE) algorithm and McCaskill's partition function of the thermodynamic equilibrium [34].

Results of this study are consistent with selection upon mRNA structures: numbers of conserved optimal codons and synonymous substitutions are reduced at structurally important sites.

## Methods

### Choice of study organism & data

I focus on *Saccharomyces cerevisiae*, as this model eukaryote is well studied, with genome sequences available for it and several related species. Importantly yeast allows using optimal codon numbers to investigate alternative selective constraints while controlling for effects of base composition. This is because (i) translational selection has been investigated extensively and supported in yeast [1-3]: certain translationally "optimal" codons increase in frequency with expression level and correspond to the most abundant tRNAs in the cell or to the tRNA with which they form the strongest binding. (ii) Crucially, translationally optimal codons in yeast are not biased towards GC-ending codons, as in many other Eukaryotic organisms. In yeast 12 optimal codons end with G or C (-GC), 12 with A or T (-AT). To control for base composition is important as RNA secondary structure predictions are – at least partly- based on thermodynamic properties and will therefore be affected by GC content: GC nucleotides form the most stable binding with three hydrogen bonds and will consequently more likely be paired in the structure. From the yeast alignments provided by Kellis et al. [35] comparing *Saccharomyces cerevisiae *with *S. paradoxus*, *S. mikatae *and *S. bayanus*, I use 492 genes that have start and stop codons but no premature stop codons or frame-shifting indels in all four species.

### Secondary structure

I predict the secondary structure of the coding sequences using the below methods and identify for each nucleotide whether it is paired with another nucleotide, or unpaired. I assume paired sites are more important for the predicted *secondary *structure than unpaired sites. Note however, that unpaired sites may well be important for maintaining the mRNA's *tertiary *structure.

### Secondary structure prediction methods

The thermodynamic stability of a secondary structure is measured as the amount of free energy released or used by forming base pairs. Positive free energy requires work to form a structure, negative free energy releases stored work. Free energy parameters are estimated from chemical melting experiments. The widely used Minimum Free Energy (MFE) algorithm [28] computes the one single structure with the most negative energy value, that thermodynamically is hence the most likely to be formed. The MFE algorithm seems fairly accurate for short RNA sequences, for which ~73% of paired sites are accurately predicted. mRNAs however are likely to be present in a population of structures [36,37]. Often 5–10% of structures share very similar free energy values [38], and the predicted MFE structure might just be one out of many thermodynamically similar structures. Taking all possible secondary structures of the thermodynamic equilibrium into account, McCaskill's algorithm [34] computes the most probable structure and calculates the probability that each site is paired. When taking base pairings with high probabilities, the accuracy of the prediction increases [29]. Another benefit of McCaskill's algorithm is that it is less affected by small but reasonable variations in the underlying energy parameters – while the MFE prediction is very sensitive [39,40]. I used the RNAfold (Vienna RNA Secondary Structure[33,41]) package to predict structures of the four yeasts separately using the MFE and McCaskill's algorithms. When using McCaskill's algorithm, I consider sites to be paired that pair with high probability (>2/3) across the structure ensemble; all other sites are considered as unpaired. With increasing sequence lengths predictive accuracy decreases presumably because of the enormous increase in the number of potential base pairings that can be made as sequence length increases [42]. I therefore look at both the complete set of genes, and at the subset of genes shorter than 800 bp.

To predict the secondary structure, one can also assume structural conservation, and compute the one consensus structure that allows the largest amount of structural conservation across homologous sequences. Especially supportive of structural conservation are sites that vary at the sequence level but retain potential of Watson-Crick pairings in the structure (co-variations). Structures predicted with the aid of comparative data appear to be more accurate than those based on thermodynamic properties alone [30-32]. I use the ALIfold package [33,43] that integrates comparative information in the prediction made with either MFE or McCaskill's algorithm and predict the consensus structures of the four yeasts together using the ALIfold default settings for co-variation weight (Φ_1 _= 1, and Φ_2 _= 1).

### Optimal codon use

Codon identification is based on the *S. cerevisiae *sequence. Optimal codons are defined as in Kliman et al. (2003) [44]. The relative frequency of optimal codons (F_op_[45]) is the ratio of optimal codons to synonymous codons. I compute the relative frequency of optimal codons for each amino acid and gene separately. For amino acids with both one AT- as well as one GC-ending optimal codon (thr, val, ile, ser), I compute the relative optimal codon frequencies of the two optimal codons per amino acid separately. Throughout the paper, the terms "optimal" and "suboptimal" will refer to translational selection.

### Tests

If there is selection for secondary structures, one may expect higher constraint at structurally important (paired) than at structurally less important (unpaired) sites.

(1) Under translational selection one may expect lower numbers of translationally optimal codons at paired compared to unpaired sites. Note that the analysis is restricted to those codons that are conserved across the four yeast species and are likely to experience stronger selection pressures. Restricting the analysis to conserved sites is crucial for the ALIfold measure, as it incorporates substitutions in its prediction: ALIfold may tend to pair conserved sites, and under translational selection conserved sites tend to have higher optimal codon use than non-optimal sites. This could generate an artificial positive correlation between optimal codon numbers and structure when considering all codons. As GC-ending optimal codons are more likely to be paired, I look at GC- and AT-ending optimal codons separately. I do this for the four yeast species separately (using RNAfold) as well as for their consensus structure (using ALIfold), using MFE as well as McCaskill's algorithm for both methods.

(2) If mRNA structures are conserved across species one may further expect lower numbers of substitutions at paired compared to unpaired sites. As ALIfold incorporates comparative information, this test is only meaningful for structures predicted by RNAfold. Codons experiencing non-synonymous substitutions are excluded from this analysis as one may expect possible selection on mRNA structure will mainly affect synonymous substitutions, while non-synonymous substitutions will be more constrained for other reasons. To check the structural similarity and potential conservation of predicted structures across species, I first compute the relative number of base pairings per gene that are consistently, i.e. unambiguously, predicted to be paired or unpaired across species. To estimate structural constraint at synonymous sites, I count for each synonymous optimal and non-optimal codon how often the respective third codon position is paired and unpaired in the *S. cerevisiae *structure (RNAfold) and how often the codon is conserved or experiences a synonymous substitution compared to *S. paravensis*. Note that translational selection and structural selection may be counter-balancing with respect to synonymous substitution numbers. I.e. unpaired sites with high numbers of optimal codons may experience reduced synonymous substitution numbers due to translational selection while paired sites with high numbers of non-optimal codons may experience reduced synonymous substitution numbers due to structural selection. To disentangle structural selection from translational selection, I look at optimal and non-optimal codons separately as. I further look at GC- and AT-ending codons separately as mutational processes and gene conversion events may be compositionally biased [46].

### Statistics

Each of our analyses generates a set of 2 × 2 contingency tables per gene and per amino acid or codon. These are divided according to whether the site is paired or unpaired in the predicted secondary structure, and whether (1) the codon is optimal or non-optimal, and whether (2) the codon is conserved or synonymous polymorphic across the four species. To combine these independent 2 × 2 tables, I use the Mantel-Haenszel Z statistic according to Sokal and Rohlf [47]. I compute joint probabilities for all tables or certain subsets. To disentangle an effect of GC content on synonymous codon use at paired sites, I combine amino acids with AT-ending ending and amino acids with GC-ending optimal codons. I exclude contingency tables when expected values were zero, tested for homogeneity and computed the joint odds ratio (W_MH_) and its significance, including the continuity correction. I orient the odds ratio such that selection in favour of mRNA secondary structure is indicated by W_MH _<1: i.e. lower numbers of optimal codons, and lower numbers of synonymous substitutions at paired sites.

## Results

1) Conserved optimal codon numbers are significantly lower at paired compared to unpaired sites, irrespective of the method (RNA- and ALIfold) and algorithm (MFE and McCaskill's) used to predict secondary structure. Crucially, the tendency remains whether I consider amino acids with AT- or GC-ending optimal codons (Tables 1, 2).

**Table 1:** Comparison of conserved optimal codon numbers at paired and unpaired sites.

Method	Algorithm	ALL	GC-ending	AT-ending	GC leu & lys
**Genes shorter than 800 bp**					

RNAfold *S. cerevisiae*	MFE	0.646 ***	0.624 **	0.503 ***	1.353 ***
	Mc	0.542 ***	0.567 ***	0.422 ***	0.910 ***
RNAfold *S. paravensis*	MFE	0.667 ***	0.608 ***	0.542 NS	1.137 ***
	Mc	0.560 ***	0.571 ***	0.407 **	1.119 ***
RNAfold *S. mikitae*	MFE	0.653 ***	0.590 ***	0.544 *	1.172 ***
	Mc	0.572 ***	0.623 ***	0.411 ***	1.131 ***
RNAfold *S. bayanus*	MFE	0.640 ***	0.597 **	0.502 ***	1.181 ***
	Mc	0.537 **	0.557 ***	0.401 ***	1.003 ***
ALIfold	MFE	0.638 ***	0.577 ***	0.465 NS	1.499 ***
	Mc	0.468 **	0.444 ***	0.326 ***	0.997 ***

**All genes**					

RNAfold *S. cerevisiae*	MFE	0.920 ***	0.863 ***	0.751 ***	1.584 ***
	Mc	0.841 ***	0.866 ***	0.676 ***	1.436 ***
RNAfold *S. paravensis*	MFE	0.878 ***	0.826 ***	0.733 ***	1.497 ***
	Mc	0.819 ***	0.822 ***	0.659 ***	1.460 ***
RNAfold *S. mikitae*	MFE	0.912 ***	0.814 ***	0.790 NS	1.160 ***
	Mc	0.839 ***	0.869 ***	0.740 NS	1.404 ***
RNAfold *S. bayanus*	MFE	0.904 ***	0.855 ***	0.742 ***	1.590 ***
	Mc	0.833 ***	0.840 ***	0.675 ***	1.455 ***
ALIfold	MFE	0.899 ***	0.759 **	0.739 NS	1.937 ***
	Mc	0.770 ***	0.724 ***	0.645 ***	1.529 ***

I combine contingency tables for all amino acids and genes (ALL) and subsets of amino acids with GC- and AT- ending optimal codons (leu and lys are treated separately, as these two GC-ending amino acids behave very opposing, see below Table 2). Mantel Haenzsel estimators and significances are presented, W_MH _<1 = lower optimal codon use at paired than at unpaired sites.

* < 0.05, ** < 0.01, *** < 0.005, NS = not significant. Structure prediction is based on ALIfold and RNAfold using MFE and McCaskill's (Mc) algorithm.

**Table 2:** Comparison of conserved optimal codon numbers at paired and unpaired sites.

	RNAfold (*S. cerevisiae*)		ALIfold	
		
	MFE	Mc	MFE	Mc
**Amino acids with GC-ending optimal codons**				

**Leu**_**TTG**_	1.380 ***	1.273 ***	1.811 ***	1.456 ***
**Lys**_**AAG**_	1.772 ***	1.654 ***	2.019 ***	1.654 ***
**Phe**_**TTC**_	1.075 ***	1.120 ***	0.786 NS	0.864 **
**Tyr**_**TAC**_	0.809 NS	0.720 NS	0.741 NS	0.631 NS
**His**_**CAC**_	0.707 NS	0.600 NS	0.568 NS	0.657 *
**Asp**_**GAC**_	0.813 NS	0.838 NS	0.656 ***	0.746 **
**Asn**_**AAC**_	0.891 *	0.831 NS	0.725 **	0.637 NS

**Amino acids with one GC- and one AT-ending optimal codon**				

**Ile**_**ATC**_	1.318 ***	1.254 ***	1.555 ***	1.049 ***
**Ile**_**ATT**_	1.272 ***	0.978 ***	1.561 ***	1.009 ***
**Val**_**GTC**_	0.585 NS	0.852 ***	0.734 ***	0.560 ***
**Val**_**GTT**_	0.705 *	0.794 NS	0.806 *	0.686 NS
**Thr**_**ACC**_	0.838 **	0.921 ***	0.811 ***	0.750 ***
**Thr**_**ACT**_	0.940 ***	0.876 ***	0.932 ***	0.655 ***
**Ser**_**TCC**_	0.614 NS	0.668 NS	0.583 NS	0.591 NS
**Ser**_**TCT**_	0.794 NS	0.816 NS	0.816 ***	0.985 ***

**Amino acids with AT-ending optimal codons**				

**Ala**_**GCT**_	0.968 **	0.904 ***	1.073 ***	0.883 ***
**Arg**_**AGA,CGT**_	0.588 ***	0.545 ***	0.518 ***	0.460 ***
**Gly**_**GGT**_	0.894 NS	0.779 NS	0.901 ***	0.752 NS
**Gln**_**CAA**_	0.442 ***	0.353 ***	0.293 ***	0.278 ***
**Glu**_**GAA**_	0.946 ***	0.350 ***	0.386 ***	0.315 ***
**Pro**_**CCA**_	0.851 NS	0.734 NS	0.729 **	0.689 *
**Cys**_**TGT**_	0.500 NS	0.382 NS	0.755 ***	0.403 NS

Separately for each amino acid, I combine contingency tables of the different genes. Mantel Haenzsel estimators and significances are presented, with W_MH _<1 = lower optimal codon use at paired than at unpaired sites. Structure prediction is based on ALIfold and RNAfold using MFE and McCaskill's (Mc) algorithm.

* < 0.05, ** < 0.01, *** < 0.005, NS = not significant

The tendency is true for most amino acids separately; even GC-ending optimal codons that are more likely to be paired for thermodynamic reasons tend to be less frequent at paired sites (Table 2). Notable exceptions however are leu, lys, ile, and for RNAfold additionally phe (Table 2). One explanation for these exceptions may be that selection strength for translationally optimal codons is stronger in these amino acids, for example translational errors may be more likely or more costly. Considering prediction accuracy may decrease with gene length, I first restricted the data to genes shorter than 800 bp; including all genes however does not change the result.

(2) I first check the similarity and potential conservation of structures predicted by RNAfold. The major parts of mRNAs do not seem conserved in structure across species or prediction accuracy is low: 75% of sites are ambiguous, i.e. predicted to be paired in one or more species, but predicted to be unpaired in the remaining species (Table 3). When looking pairwise on average 41% of sites are ambiguous; number of ambiguous sites is only slightly lower for short genes. The ambiguity of predicted structural status will introduce considerable noise and may cause non-significant results. Despite high ambiguity in structure prediction the numbers of synonymous substitutions are consistently lower (W_MH_<1) at paired sites (Table 4). The tendencies remain when restricting the data to genes shorter than 800 bp. Other species comparisons lead to similar results (data not presented). Results become significant for GC-ending (optimal and non-optimal) codons (when structure is predicted using McCaskill's algorithm). G and C nucleotides do not only form stronger bonds and are more likely to be paired and structurally important, they are also more likely to be unambiguously predicted paired than A and T nucleotides (means GC: 0.263, AT: 0.153, t = 29.8409, df = 866.05, ***). This could reduce the level of noise and cause the significance of results for GC-ending codons.

**Table 3:** Similarity of predicted structures for species pairs.

Species comparison	Prediction Method	(P+U)/all	P/all	U/all
**Genes shorter than 800 bp**				

**Across all 4 yeasts**	MFE	27% ± 0.6	17% ± 0.3	10% ± 0.5
	Mc	27% ± 0.4	9% ± 0.2	18% ± 0.3
***S. cerevisiae – S. paravensis***	MFE	63% ± 0.6	36% ± 0.3	27% ± 0.3
	Mc	64% ± 0.5	22% ± 0.4	42% ± 0.5
***S. cerevisiae – S. mikitae***	MFE	59% ± 0.3	34% ± 0.2	25% ± 0.2
	Mc	61% ± 0.4	20% ± 0.4	41% ± 0.6
***S. cerevisiae – S. bayanus***	MFE	42% ± 0.4	23% ± 0.5	19% ± 0.1
	Mc	59% ± 0.3	20% ± 0.0	40% ± 0.5

**All genes**				

**Across all 4 yeasts**	MFE	25% ± 0.3	8% ± 0.1	16% ± 0.2
	Mc	25% ± 0.4	16% ± 0.2	9% ± 0.1
***S. cerevisiae – S. paravensis***	MFE	61% ± 0.4	36% ± 0.2	25% ± 0.2
	Mc	63% ± 0.3	22% ± 0.3	41% ± 0.4
***S. cerevisiae – S. mikitae***	MFE	58% ± 0.2	35% ± 0.2	23% ± 0.1
	Mc	60% ± 0.3	20% ± 0.3	40% ± 0.4
***S. cerevisiae – S. bayanus***	MFE	57% ± 0.2	34% ± 0.1	23% ± 0.1
	Mc	58% ± 0.2	20% ± 0.2	39% ± 0.4

The average percentages of sites (± variances) unambiguously predicted to be paired (P/all) and/or unpaired ((P+U)/all, U/all) for the respective species comparison using RNAfold MFE and McCaskill's (Mc) algorithm are presented.

**Table 4:** Comparison of synonymous substitution numbers at paired and unpaired sites.

		**AT**_**Opt**_	**AT**_**Nopt**_	AT	**GC**_**Opt**_	**GC**_**Nopt**_	GC	All
**(1)**	**MFE**	0.511 NS	0.542 NS	0.600 NS	0.488 NS	0.296 NS	0.414 NS	0.481 NS
	**Mc**	0.567 NS	0.528 NS	0.546 NS	0.544**	0.327*	0.458**	0.505 NS
**(2)**	**MFE**	0.255 NS	0.201 NS	0.232 NS	0.140 NS	0.257 NS	0.213 NS	0.225 NS
	**Mc**	0.255 NS	0.230 NS	0.238 NS	0.264 NS	0.167 NS	0.222 NS	0.232 NS

Looking at *S. cerevisiae *and *S. paravensis *, I compare numbers of each codon in *S. cerevisiae *being either synonymous non-conserved or conserved at paired or unpaired sites. Structure prediction is based on RNAfold upon the *S. cerevisiae *sequence using MFE and McCaskill's (Mc) algorithm. Mantel Haenzsel estimators and significances are presented. W_MH_<1 = lower numbers of synonymous substitutions at paired sites. (1) All genes, (2) Genes that are shorter than 800 bp.

* < 0.05, ** < 0.01, *** < 0.005, NS = not significant

## Discussion

I tested for evidence of selective constraint acting on mRNA secondary structures in protein coding yeast genes. Predicted secondary structures differ greatly according to the prediction method used and between species. Nevertheless, there are significantly fewer conserved optimal codons and consistently fewer synonymous substitutions at paired sites for all predicted secondary structures. The results of this study are consistent with purifying selection on mRNA secondary structures.

Similar tendencies of codon use have been reported for Drosophila and humans: mRNA stability seems high when optimal codon use is low in Drosophila [48] and paired sites contain an excess of rare codons in humans [49]. Note that in this study, the comparison of optimal codon use is restricted to conserved sites. Besides the methodological need for ALIfold (see Material and Methods), the restriction to conserved sites restricts the analysis to sites potentially under considerable strong selection. For RNAfold structures, results become non-significant when not restricting the data to these conserved sites (data not presented). Strong conflicting selection pressures seem to act on certain sites while the remaining sites seem less constrained for structure. Selection on *local *and not *global *structures may explain these results and contribute to the low structural similarity across species. Selection on local mRNA structures in coding regions of eukaryotic genes has been suggested before [49]. Beside the low structural similarity also compensatory substitutions may contribute to the non-significant results when comparing substitution numbers at paired and unpaired sites.

Previous bioinformatic studies that focussed on whether or not the thermodynamic *stability *of mRNA structures of various organisms is selected for or against [20-23,49,55] lead to partly inconsistent results and controversies about the accurate randomization procedure. In these studies, the observed MFE is compared to the expected MFE, which is estimated by taking the mean MFE of randomized versions of the same sequence, and a significant deviation is taken as evidence for selection for or against thermodynamic stability of the structure. The randomization of sequences can be performed in a number of different ways holding various properties of the sequence constant, while randomizing others. The properties are of biological importance; variables that are affected by forces other than selection for mRNA structure – for example the amino acid sequence – should be fixed. Which variables should remain free to vary however may not always be obvious, while the results are very sensitive to them. Di-nucleotide content for example might be selected for its effect on stability and should be allowed to vary for randomized sequences argue Chamary and Hurst [22]. However, di-nucleotides might well be affected by mutation bias, or selected for some other reason [21], in which case, di-nucleotide content should be kept fixed. The control of di-nucleotides in fact renders significant results non-significant [20-23,55].

Note that in contrast to comparing observed and expected MFE values, the comparison of constraint at paired and unpaired sites does not indicate that selection acts for or against the thermodynamic *stability *of the structure, but that the very predicted *structure *is under selection. With respect to selection for or against *stability *of structures, ALIfold results indicate that the thermodynamically most stable global structure is not conserved across the four yeasts: ALIfold consensus energy value is much higher i.e. less stable compared to the average energy value of the single sequences [see also Washietl et al. [50] for approach]. This is conform with results of Babak et al. [24] which support selection against stability of structures in coding regions. It is reasonable to expect selection on mRNA structures may act against too stable structures because too stable and un-flexible mRNA structures may interfere for instance with translation [18] and some mRNAs flexibility may allow their specific and dynamic complexes with other factors. mRNAs lead a complex life [51] and besides thermodynamic stability, selection on mRNA structure may also exist to maintain specific local or global mRNA structures that allow binding and interaction with other factors and thus effect biological functioning. Not only structural targets may be of effect, also accessibility of sequence targets may depend on global or local mRNA structures.

While results of this study are consistent with selection upon mRNA structures in coding regions and support laboratory studies that report synonymous substitutions are functionally important with respect to mRNA structure and translation in humans [52-54], two considerations should be made. First, we do not know whether thermodynamic mRNA structure predictions predict the mRNA structures that are formed in the cell. mRNAs are generally associated with other factors [51], and effects of mRNA-associated microRNAs and proteins on the structure are hard to predict. Also, kinetics of mRNA folding and pseudo-knots are not considered here. Even with the comparative method, mRNA structures may remain at best approximations of the real mRNA structures in the cell. Secondly, the predicted and also the real structure will be affected by certain DNA patterns – however whether or not the respective DNA patterns are selected for mRNA structure or another reason may be hard to judge. There are several DNA patterns one may consider. (i) Di-nucleotide content of naturally occurring sequences leads to higher than expected thermodynamic stability [e.g. [21,23,55]]. Di-nucleotide content may be selected for its effect on mRNA structure but it may also be affected by mutation bias, or selected for some other reason, for example for nucleosome positioning [56-58] or transcription pause sites [59]. (ii) Frequency of polypurine tracts is increased in exons and may affect thermodynamic structure. Again, polypurine tracts may be selected with respect to mRNA structure but also for other reasons such as enhancing splicing [60]. (iii) Translational protein folding into alpha-helix and beta sheets may affect synonymous codon use [61] and periodic DNA patterns may affect mRNA structure. If thermodynamic predictions correspond to any other force such as selection on nucleosome positioning and transcription or co-translational pause sites, the observed patterns may be a consequence of that and inference of selection acting directly upon on mRNA structure may be incorrect.

Alternative selection upon mRNA structures (or any other selective target) may counterbalance translational selection and explain why the bias towards translationally optimal codon is never complete and even in highly expressed genes non-optimal codons are used. Alternative selection may also contribute to the discrepancy between expected and observed codon bias [58], and may lead to systematic underestimates of selection strength for optimal codons. As selection for mRNA structures may be acting stronger on GC-ending codons, in organisms in which potential translationally optimal codons are biased towards GC, such as Drosophila, mammals, C. elegans, estimates of selective strength for optimal codons may also be overestimated. It will be worth considering effects of alternative selection and disentangling the different targets of selection.

## Conclusion

I tested for evidence of selective constraint acting on mRNA secondary structures in yeast. Predicted structures differ greatly according to the prediction method used and between species. Nevertheless, there are significantly fewer conserved optimal codons and consistently fewer synonymous substitutions at paired sites for all predicted secondary structures. These results are consistent with purifying selection on mRNA secondary structures in protein coding yeast sequences and suggest their biological importance. One should consider however that accuracy of structure prediction is unknown for mRNAs and interrelated selective forces may contribute. Selective pressures alternative to translational selection seem affect synonymous and optimal codon use in yeast. Depending on strength and direction of translational selection in an organism, such alternative selective forces may lead to under- or over-estimates of selective strength on optimal codon use.
